# Incidental pulmonary findings on CT in daily practice: the nodule and the interstitial lung abnormalities - what’s old, what’s new

**DOI:** 10.1007/s11547-025-02134-4

**Published:** 2025-11-26

**Authors:** Giorgio Maria Masci, Luca Giuliani, Roberto Romiti, Michele Massaro, Cosimo Nardi, Flaminia De Cristofaro, Valeria Panebianco, Carlo Catalano, Nicholas Landini

**Affiliations:** 1 Department of Radiological, Oncological and Pathological Sciences, Policlinico Umberto I, Sapienza University of Rome, Viale del Policlinico 155, 00161 Rome, Italy; 2 Department of Internal Medicine and Atherosclerosis Prevention, Policlinico Umberto I, Sapienza University of Rome, Viale del Policlinico 155, 00161 Rome, Italy; 3 Department of Biomedical, Experimental and Clinical Sciences “Mario Serio”, University of Florence, Viale Morgagni 50, 50134 Florence, Italy

**Keywords:** Incidental findings, Solitary pulmonary nodule, Interstitial lung diseases, Interstitial lung abnormalities, Computed tomography, Practice guidelines as topic

## Abstract

The growing use of computed tomography (CT) in clinical practice has led to an increase in incidental pulmonary findings, with nodules being among the most commonly encountered. Managing these nodules remains a significant challenge in clinical radiology. In addition to nodules, interstitial lung abnormalities (ILAs) have emerged as a newly defined entity, necessitating recognition to prevent underestimation and misinterpretation, as well as to guide appropriate management. This review aims to examine the interpretation of incidental pulmonary nodules, providing clarity on their management and addressing gaps not covered by the 2017 Fleischner Society Guidelines. Additionally, we focus on recent updates related to ILA classification, as outlined by the Fleischner Society, and highlight key radiologic features critical for distinguishing ILAs from non-ILA alterations. Finally, we explore the potential future developments in the evaluation of ILAs, offering insights into how the radiologists' role in managing these abnormalities may evolve.

**Graphical abstract:**

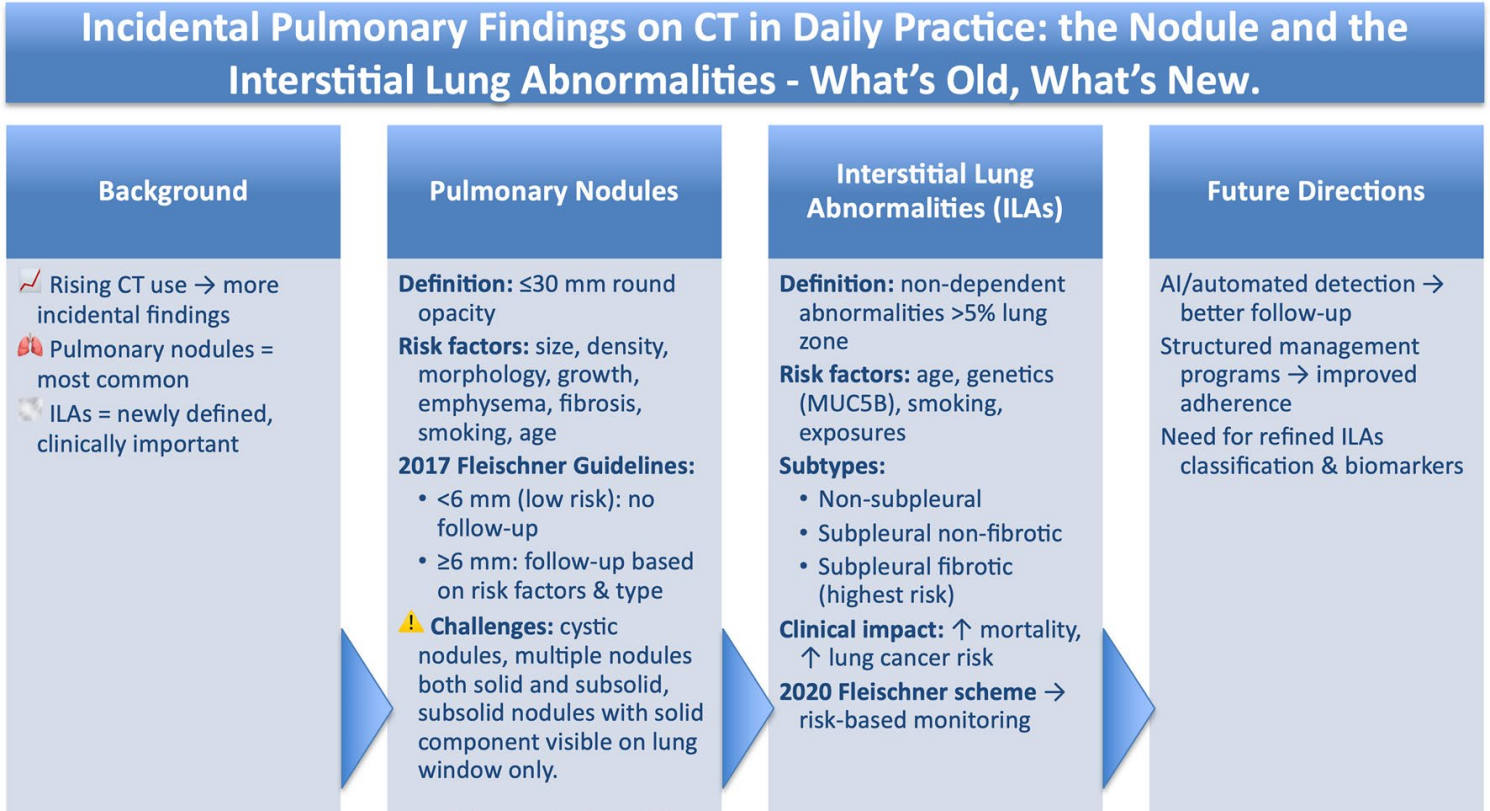

## Introduction

An incidental finding (IF) is defined by the American College of Radiology as “an incidentally discovered mass or lesion, detected by CT, MRI, or other imaging modality performed for an unrelated reason” [1]. This definition can be easily extended to any abnormal finding seen in a radiological examination which is independent to the original clinical inquiry.

Computed tomography (CT) is a potentially pan-exploring investigation, and its demand has been increasing over years, thus increasing the likelihood of discovering incidental findings [2]. CT of the thorax is largely demanded and may be performed as a first or second level imaging examination. In addition, thorax is scanned in total body CT, while neck and abdominal CT scans may include the upper or lower zones of the lungs, respectively. Hence, having to deal with a thoracic incidental finding is not uncommon.

Although a wide spectrum of entities may be encountered in thoracic scans, this review focuses on two particular incidental findings that may undermine radiologists when taking a stand concerning their correct management: the pulmonary nodule, which is probably the most common and discussed pulmonary finding in CT, although the available guidelines were published some time ago, and the interstitial lung abnormalities (ILAs), an entity of recent relevance which is defined by the Fleischner Society as CT findings potentially compatible with interstitial lung disease (ILD) in patients undergoing partial or complete chest CT examination without previous clinical suspicion of ILD [3]. Aim of this paper is to provide a valid support for radiologists for the interpretation of these IF and their management, clarifying equivocal situations and raising issues that, as a result of new evidence, evolving needs, or the recent conceptualization that is still not fully consolidated, may require further discussion.

## Pulmonary nodule

A pulmonary nodule was defined by the Fleischner Society as a typically round opacity, less than or equal to 30 mm in average diameter [4]. Pulmonary nodules may be discovered incidentally in about 30% of chest CT scans and, with the increasing number of performed chest examination, detection of incidental pulmonary nodule (IPN) is steadily increasing [5, 6]. In 2017, the Fleischner Society released the latest guidelines for management of non-calcified IPN with the purpose of reducing the number of unnecessary follow-up examinations [7]. In particular, the target was to avoid the follow-up of nodules with less than 1% probability of malignancy, providing greater discretion to the radiologist, clinician, and patient to make management decisions. The most significant modifications provided compared to the original version [8] include the consideration of subsolid and multiple nodules in the management algorithm and the increase of the minimal threshold size for follow-up from 4 to 6 mm [9]. These recommendations apply to adults who are at least 35 years old, since younger patients are considered to have a low risk for malignancy [10, 11]. It is important to know that the Fleischner Society guidelines for the management of IPN should not be used in the following scenarios: a) in the setting of lung cancer screening programs, for which Lung-RADS criteria must be considered [12], since they are specifically designed for high-risk individuals; b) in patients with a recent history of malignancy (higher risk of metastatic disease); c) in immunocompromised patients, as opportunistic infections are more probable in these individuals [13]. Fleischner guidelines take into account a spectrum of variables related both to nodule’s characteristics and to patient’s risk for lung primary malignancy, which are based on estimations of the individual risk of malignancy and prediction models [7] (Table 1).

**Table 1:** Risk factors for malignancy (summarized and adapted from Ref. [8])

Related to the nodule	Comments
Size	Increase in size is directly related with the risk of malignancy
Density	Solid or part-solid nodules are associated with an increased risk of malignancy-aggressiveness compared to ground glass nodules; calcifications or fat within a nodule may be suggestive of a benign nodule
Morphology	Spiculated and irregular margins are associated with an increased risk of malignancy
Location	Primary lung cancer has predilection for upper lobes, while metastases for the periphery and lower lobes
Number	To be evaluated together with all other characteristics
*Related to lung parenchyma*	
Emphysema	Associated with increased risk of lung cancer
Fibrosis	Associated with increased risk of lung cancer
*Related to the patient*	
Age	After 35–40 years, risk of lung cancer increases steadily after every decade
Sex	Female gender is associated with a higher risk of lung cancer
Race	Black men have an increased risk of lung cancer compared to white men
Family history	A family history of lung cancer increases the risk of 1.5 times
Smoking history	Cigarette smoking increases the risk of lung cancer from 10 to 35 times

### Risk factors related to the nodule

*Size.* There is substantial evidence that size is directly related to the probability of nodule’s malignancy [14]. In a review conducted by Wahidi and colleagues, the prevalence of malignancy in nodules that measured < 5 mm was exceedingly low (range, 0 to 1%), while the risk for malignancy was higher in nodules that measured between 5 and 10 mm (range, 6–28%), and it was very high in nodules that measured > 2 cm in diameter (range, 64–82%) [15].

However, to our knowledge, the size modification over time of a nodule remains the most important factor to define the risk of malignancy of nodule. In 1994, Usuda firstly introduced the volume doubling time (VDT) of tumors detected on chest X-ray films, defined as the number of days in which a nodule doubles its volume [16]. Since then, VDT has been applied to CT for discriminating between nodules that are infectious/inflammatory malignant and benign. For instance, regarding solid nodules, a VDT at risk for malignancy typically ranges from 20 to 400 days, while less than 20 days may suggest an inflammatory condition and more than 400 days is helpful to identify benignant lesions [17–19]. However, it is worth mentioning that ground glass nodules may have a VDT > 400, still representing foci of atypical adenomatous hyperplasia with potential of malignancy or even bronchioloalveolar carcinoma [20].

*Density.* The nodules can be distinguished based on its attenuation in solid, when they show a soft tissue attenuation, or subsolid, when they have a ground glass density or both components, the latter defined part-solid nodules [4].

**Fig. 1:**
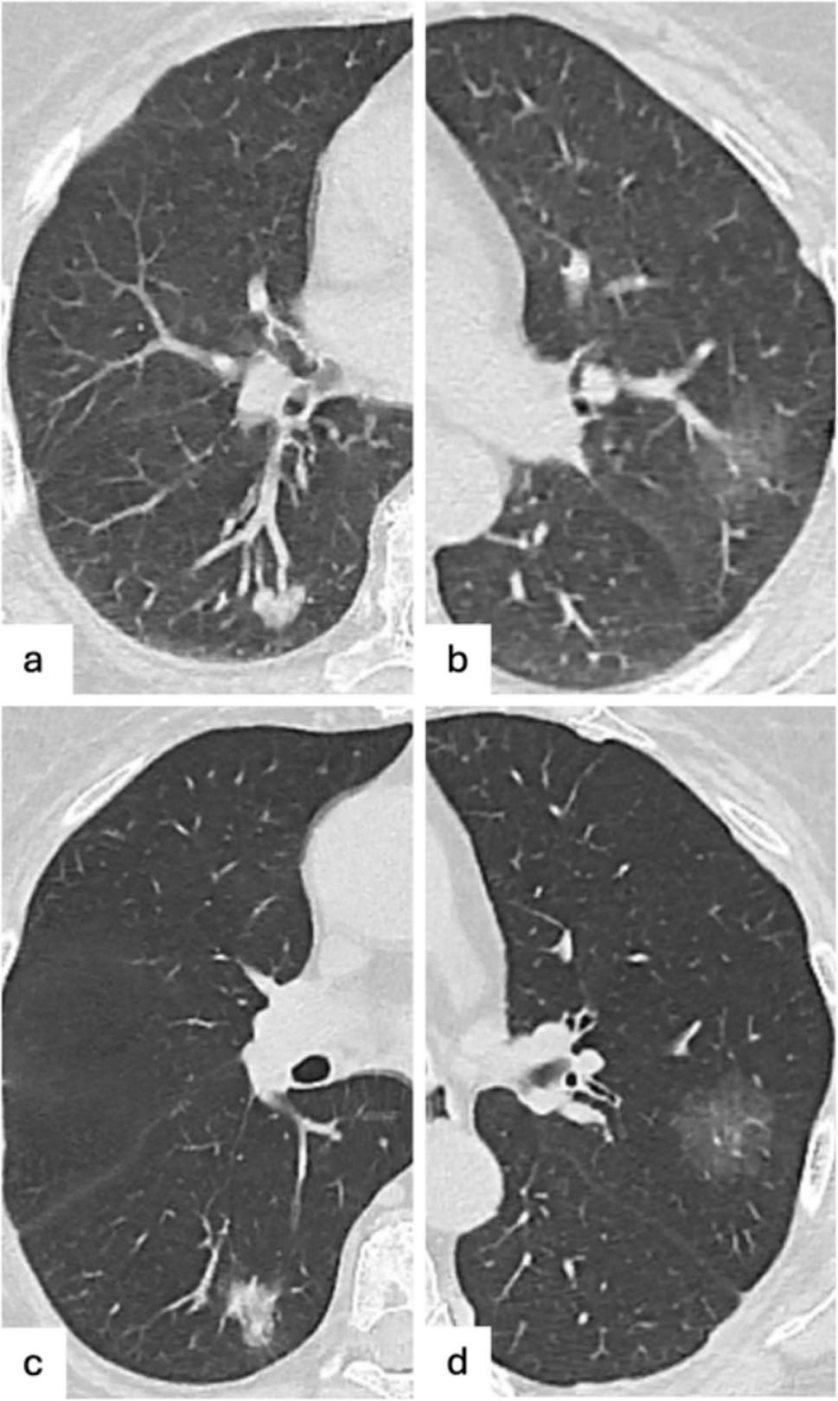
Seventy-two-year-old patient with a part-solid nodule in the right lower lobe (**a**) and a pure ground glass nodule in the left upper lobe (**b**). After 1 year, the part solid nodule increased in size (**c**) and the ground glass nodule remained stable (**d**). Both nodules were resected resulting in an invasive adenocarcinoma (part-solid nodule) and an adenocarcinoma in situ (ground glass nodule)

Overall, part solid nodules have been associated to an increased risk of primary lung malignancy compared with solid nodules, whereas pure ground glass nodules were considered with a reduced risk of malignancy than solid nodules [14]. It has been clarified that pure ground glass opacity represents a significant prognostic factor of less invasive cancer (atypical adenomatous hyperplasia or adenocarcinoma in situ), while the presence of a solid component corresponds to tumor invasion and, therefore, represents a predictor of malignancy [21–23]. A recent prospective multicenter study showed that the majority of prognostically significant lung cancers did not derive from the SSN, but arose in a different area of the lung [24] (Fig. 1), suggesting a more conservative approach of SSN based on active surveillance in order to avoid unnecessary surgery, preserving the lung function in subjects that subsequently required resection of more aggressive cancer in other pulmonary sites. Additional features that may be related to the risk of malignancy in ground glass nodules and that may be useful in their interpretation have been investigated, as inner low attenuation areas (bubble-like lucencies), well-defined peripheral patch or “halo sign” (as opposed to ill-defined halo sign found in inflammatory nodules), inner air bronchogram and or associated distortions of vessels and airways [25, 26].

**Fig. 2:**
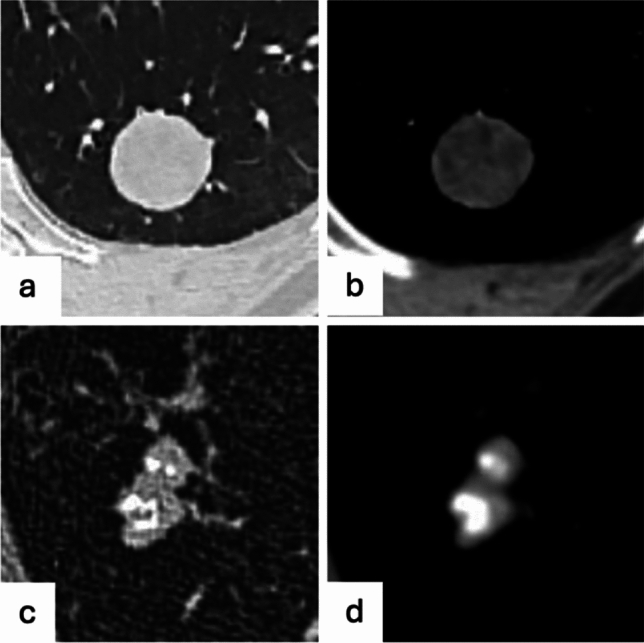
A round, well-defined nodule (**a** and **b**) with areas of intralesional fat seen in the soft tissue window, suggesting a pulmonary hamartoma and a lobulated, well-defined nodule with central “popcorn”-like calcification, also indicative of pulmonary hamartoma (**c** and **d**)

Higher-attenuation areas within a nodule may represent calcification, which are generally indicative of a benign etiology [27]. Calcification is usually considered as a region of > 175 Hounsfield Units (HU), but nodules with areas of 40 to 175 HU on non-contrast CT may contain microscopic calcifications. Diffuse calcified nodules or those with measurable central calcification typically represent granulomas from previous infections, while a “popcorn”-like calcification is suggestive of benign pulmonary hamartoma (Fig. 2 and d), even if the fatty component is not detectable, while eccentric or irregular calcifications may raise the suspicion of malignancy [28]. Furthermore, it is worth to remind that in the oncological setting and when multiple calcified nodules are presents, those may represent calcified metastases, as seen in sarcomas, papillary and mucinous carcinomas [29].

The presence of fat (ranging from − 40 to − 120 HU) in a nodule is typically indicative of benign lesion, usually hamartoma (Fig. 2a and b) or less frequently lipoma [27], however lung metastases from liposarcoma may appear as fat-containing nodules [30].

*Margins and morphology.* The presence of irregular borders (spiculation, lobulation) has been associated with an increased risk of primary lung cancer [14, 27, 31]. Conversely, round shape and smooth margins are considered to be low risk factors [32]. Perifissural nodules, when polygonal or oval in shape, found below the level of the carina and within 15 mm from the pleura, are highly suggestive of intrapulmonary lymph nodes and do not require follow-up [7, 33]. However, radiologists have to be aware that lung metastases typically appear as round variable-sized nodules, therefore in oncological patients the presence of multiple round nodules is highly suggestive for metastases [34]. Therefore, morphologic characteristics of a nodule represent weaker predictors of nodule malignancy compared to size, growth, and density [27].

*Location and distribution.* Although there is no existing rule for establishing the nature of a nodule based on its location within the lung parenchyma, it is true that some entities have predilection for specific lung zones. Previous studies based on low-dose CT screening programs proved that primary lung cancer is more commonly encountered in the upper lobes, with adenocarcinoma being more frequent in the periphery of the lung while squamous cell carcinoma found more often near the hila [14, 35, 36]. Conversely, lung metastases are more likely to occur in the periphery and in the middle and lower zones of the lung rather than in the apices, probably due to distribution of blood flow within the lung parenchyma [37–39]. However, it is important to emphasize that several non-cancerous entities may have predilection for both upper and lower lobes (i.e. infection, granulomatous diseases, rheumatoid nodules, arteriovenous malformation, exc.), therefore correlation with clinical and laboratory findings remains crucial. As previously mentioned, subpleural polygonal nodules located below the carina are typically indicative of intrapulmonary lymph nodes; however, pleural traction represents a feature of lung adenocarcinoma infiltration, helping to differentiate subpleural malignant nodules from intrapulmonary lymph nodes [9, 40]. Hence, it is important to consider location together with morphology. Based on distribution, multiple centrilobular nodules usually reflect an airway spread of infections (i.e., infection, vasculitis, neoplasm), but perilymphatic distribution, in the setting of oncologic patients, may be due to lymphangitic carcinomatosis, as well as a random distribution may represent the expression of metastatic hematogenous spread [41].

*Number.* The presence of multiple nodules was associated with a decreased likelihood that a particular nodule will be a primary pulmonary malignancy. Many benign processes (e.g. infections, sarcoidosis) may manifests with multiple nodules. Moreover, clustered nodules may be indicative of infections [27]. However, radiologists have to be aware that the mean number of lung nodules may be higher in patients with lung cancer and that multiple ground glass nodules may represents synchronous tumors. Moreover, lung cancers may have satellite nodules [42]. Lastly, multiple lung nodules in lung bases could be expression of metastases from an unknown cancer in a different site, as already highlighted. Hence, we suggest always considering the number of nodules together with all the other aforementioned characteristics.

### Risk factors related to lung parenchyma

*Emphysema.* The presence of pulmonary emphysema represents an independent risk factor for developing lung cancer [43–45]. Emphysema is characterized by an abnormal permanent enlargement of the airspaces distal to the terminal bronchioles, accompanied by destruction of their walls [46]. These abnormalities can be caused by cigarette smoking but they are also related to genetic conditions, such as alpha-1 antitrypsin deficiency [47]. The mechanism involved in the development of lung cancer in patients with emphysema, also including those with alpha-1 antitrypsin deficiency, is based on multiple factors, such as the state of oxidative stress and airways chronic inflammation caused by exposure to tobacco or the dysfunction of mucociliary clearance resulting in DNA damage and tumor growth [48, 49]. Moreover, epigenetic changes were found to link chronic obstructive pulmonary disease (including emphysema) and lung cancer through common methylation markings and subsequent changes in gene expression, such as CDKN2A, that are induced by cigarette smoking; several pathogenic roles have also been described for matrix-degrading enzymes (particularly MMP9 and MMP1) in both emphysema and lung cancer [50]. A recent systematic review and meta-analysis conducted by Yang et al. showed that centrilobular emphysema was with an increased odds of lung cancer compared to paraseptal emphysema [51]. This is coherent with the association between centrilobular emphysema and smoking history, which was not observed in the other emphysema subtypes [52, 53]. However, these results are based on a few studies and the association between different subtypes of emphysema and risk of lung cancer must be furtherly validated. In addition, Yang and colleagues showed that the severity of emphysema, more than its subtype, was associated with the most increased odds of lung cancer [51].

*Fibrosis.* In patients with idiopathic pulmonary fibrosis (IPF), the incidence of lung cancer is increased compared to normal population [54]. The relationship between IPF and lung cancer is not fully understood. One of the hypotheses involves the role of PD-L1, which is over-expressed both in IPF and lung cancer, and leads to the suppression of the immune response and therefore contributes to the pathogenesis of these diseases [55]. More specifically, The MET tyrosine kinase receptor signaling pathway is a major regulator of cell growth and proliferation and is activated in both IPF and lung cancer in response to hypoxia, leading to increased cell proliferation and tumor growth [56]. Moreover, lung cancer is believed to be more aggressive in patients with IPF, through the activation of TGF-β by fibroblasts in pulmonary fibrosis and cancer-derived epithelial cells, promoting myofibroblasts recruitment at tumor margins, protecting them from apoptosis and allowing them to invade basement membranes facilitating tumor invasion [57].

### Risk factors related to the patient

Several patient-related factors, including age, sex, race, family or smoking history, were found to be associated with a higher risk of developing lung cancer [7]. Under the age of 40, lung cancer is unusual, but for every additional decade the risk increases steadily. Female gender has been associated with an increased risk of lung cancer compared to men, probably due to women’s smaller lung size and different airway behavior, which may increase their susceptibility to lung cancer at lower exposure to cigarette smoke [58]. A higher incidence of lung cancer and mortality rates were found in black men compared to white men. Racial differences in lung cancer outcomes have been attributed several factors, including lower socioeconomic status and increased medical comorbidities (e.g., hypertension, diabetes) among black patients, but also different possibilities of treatment access and patient-provider interactions [59]. Moreover, a family history of lung cancer has been associated with a relative risk of 1.5 both in smokers and non-smokers. Cigarette smoking represents the major risk factor for lung cancer with a 10- to 35-fold increased risk compared to non-smokers. A recent cohort study with 23 years of follow-up examined the risk of lung cancer among current smokers, categorized by pack-year (PY) smoking history, showing that lung cancer risk increases with higher pack-year smoking, but the risk plateaued at higher levels of exposure. Specifically, the study found that the risk of lung cancer was high for smokers with less than 20 pack-years, but it was 3–4 times higher for those with 20–39 and 40–59 pack-years, respectively. However, the risk did not continue to increase significantly for those with 60 or more pack-years [60].

### Management of IPN according to Fleischner society guidelines

**Fig. 3:**
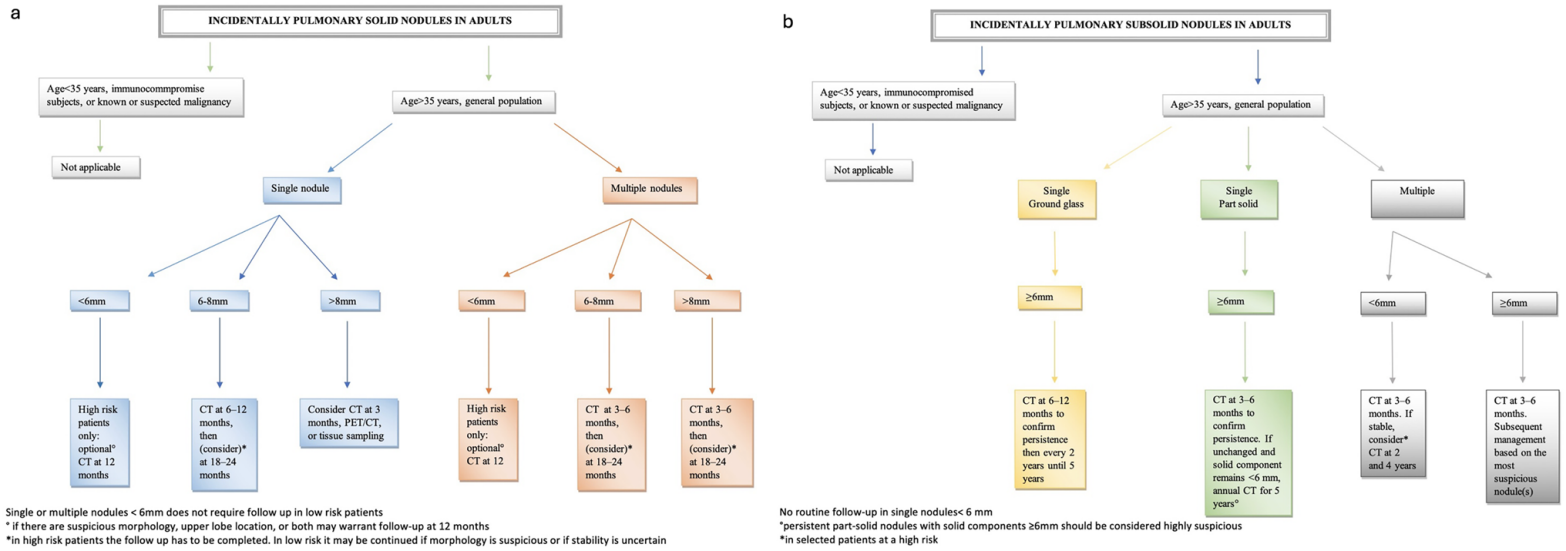
A workflow diagram derived from Fleischner Society 2017 Guidelines for Management of Incidentally Detected Pulmonary Solid (**a**) and Subsolid (**b**) Nodules in Adults. 6 mm and 8 mm as average diameter correspond to volume of 100 mm^3^ and 250 mm^3^, respectively

The 2017 Fleischner Society guidelines recommend pulmonary nodule follow-up based primarily on nodule size, density (solid or subsolid), and patient-specific risk factors. Volumetric measurements are encouraged since they provide more accurate data, but, if not available, the average of the long and short diameters should be used [7] (Table 2 and Fig. 3). A 6-mm minimal threshold is set for solid nodules or single subsolid nodules, based on a cancer risk estimate of 1% or greater [7]. Nodules smaller than 6 mm generally do not require follow-up in low-risk patients unless other high-risk features, such as spiculated or irregular margins or upper lobe location, are present, in which case a follow-up CT at 12 months may be required, with a possible second scan at 18–24 months if the nodule's morphology is suspicious [9].

**Table 2:** Fleischner Society 2017 guidelines for management of incidentally detected pulmonary nodules in adults

Solid nodules*				
	Size			
Nodule type	< 6 mm (< 100 mm^3^)	6–8 mm (100–250 mm^3^)	> 8 mm (> 250 mm^3^)	Comments
*Single*				
Low risk^†^	No routine follow-up	CT at 6–12 months, then consider CT at 18–24 months	Consider CT at 3 months, PET/CT, or tissue sampling	Nodules < 6 mm do not require routine follow-up in low-risk patients
High risk^†^	Optional CT at 12 months	CT at 6–12 months, then CT at 18–24 months	Consider CT at 3 months, PET/CT, or tissue sampling	Certain patients at high risk with suspicious nodule morphology, upper lobe location, or both may warrant 12-month follow-up
Multiple				
Low risk^†^	No routine follow-up	CT at 3–6 months, then consider CT at 18–24 months	CT at 3–6 months, then consider CT at 18–24 months	Use most suspicious nodule as guide to management. Follow-up intervals may vary according to size and risk
High risk^†^	Optional CT at 12 months	CT at 3–6 months, then at 18–24 months	CT at 3–6 months, then at 18–24 months	Use most suspicious nodule as guide to management. Follow-up intervals may vary according to size and risk

Subsolid nodules*				
	Size			
Nodule type	< 6 mm (< 100 mm^3^)	≥ 6 mm (≥ 100 mm^3^)		Comments
*Single*				
Ground glass	No routine follow-up	CT at 6–12 months to confirm persistence, then CT every 2 years until 5 years		In certain suspicious nodules < 6 mm, consider follow-up at 2 and 4 years. If solid component(s) or growth develops, consider resection
Part solid	No routine follow-up	CT at 3–6 months to confirm persistence. If unchanged and solid component remains < 6 mm, annual CT should be performed for 5 years		Part-solid nodules cannot be defined as such until ≥ 6 mm, and nodules < 6 mm do not usually require follow-up. Persistent part-solid nodules with solid components ≥ 6 mm should be considered highly suspicious
Multiple	CT at 3–6 months. If stable, consider CT at 2 and 4 years	CT at 3–6 months. Subsequent management based on the most suspicious nodule(s)		Multiple < 6 mm pure ground-glass nodules are usually benign but consider follow-up in selected patients at high risk at 2 and 4 years

Adapted with permission from reference 7

These recommendations do not apply to lung cancer screening, patients with immunosuppression, or patients with a known primary cancer

*Dimensions are the average of long and short axes, rounded to the nearest millimeter

†Consider all relevant risk factors

In case of appearance of new solid nodules smaller than 6 mm in low-risk patients, guidelines do not clarify the correct management, although this scenario may be encountered in clinical practice. In such cases, patient’s risk factors should be reassessed and if the low risk is confirmed, nodules < 6 mm are likely to be post-infection granulomas or intrapulmonary lymph nodes. However, when encountering multiple solid nodules with peripheral and basal distribution, the suspicion of metastases must be raised and follow-up at 3–6 months is required for confirmation, considering that metastatic lesions demonstrate growth within 3 months in most cases [7]. On the other hand, when encountering multiple subsolid nodules of new appearance, the inflammatory/infectious nature is probable, and a short-term follow-up CT is warranted for confirmation [31].

For multiple solid nodules that are 6 mm or larger, follow-up at 3–6 months is required and in high-risk patients an additional examination at 18–24 months is recommended. In low-risk patients the addition follow-up is optional and the decision should be based on the individual environmental risk factors of the patient, the prevalence of granulomatous infections in the geographic area, number of nodules, and morphology of the dominant nodule [9].

For ground-glass nodules 6 mm or larger, follow-up is recommended at 6–12 months and then every 2 years until 5 years. However, growth can appear even in nodules which are stable for 5 years, as previously demonstrated [61]. Hence, high-risk patients may benefit from further follow-up even after 5 years.

Part-solid nodules 6 mm or larger, with a solid component less than 6 mm in diameter, require follow-up at 3–6 months and then once a year for a minimum of 5 years, typically representing either adenocarcinoma in situ or minimally invasive adenocarcinoma [62, 63]. For part-solid nodules with solid component 6 mm or larger, a follow-up at 3–6 months is indicated for evaluating the persistence of the nodule.

For multiple part-solid nodules, with at least one nodule larger than 6 mm, management decisions should be applied to the most suspicious nodule.

### Particular circumstances

*Incidentally detected nodules on incomplete thoracic CT scans.* Part of lung parenchyma is included in CT of the neck, the heart, the spine, or the abdomen, therefore pulmonary nodules can be encountered when performing these scans. According to the latest Fleischner Society guidelines and on the basis of the estimated risk of malignancy, only nodules ≥ 6 mm require further investigation [7]. More specifically, nodules between 6 and 8 mm may benefit of a complete thoracic CT after 3–12 months, based on the clinical risk (see Table 1). No distinction is made in terms of follow‑up strategy based on the density of the nodules (solid or subsolid), meaning that management is based on risk stratification as for incidental nodules in complete chest scans. For larger nodules (> 8 mm) a complete thoracic CT scan is recommended at time of detection.

*Multiple nodules with both density patterns.* According to the Fleischner Society guidelines, nodules are strictly categorized into solid and subsolid types, even when multiple nodules are present [7]. However, in daily practice, multiple nodules with both solid and subsolid densities may be detected. This scenario appears not to be addressed by the authors, and we believe it should be clarified. While awaiting the next set of guidelines, if both solid and subsolid nodules are present in the same patient, we recommend adopting the most restrictive management approach.

*Calcified nodules.* The Fleischner Society guidelines are intended for non-calcified nodules. As mentioned, the presence of calcifications is typically associated with benign etiology and examples of typically benign nodules with calcifications are shown in the guidelines; however, eccentric or irregular calcifications are also found in primary lung cancer or in metastatic lung nodules [28], and this eventuality is not clarified. Current Lung-RADS specified that only complete calcified nodules or central, popcorn, or concentric ring calcifications may be considered benign [12]. We suggest to take into account the clarification of Lung-RADS and we expect that in next guidelines this aspect will be better defined, too.

**Fig. 4:**
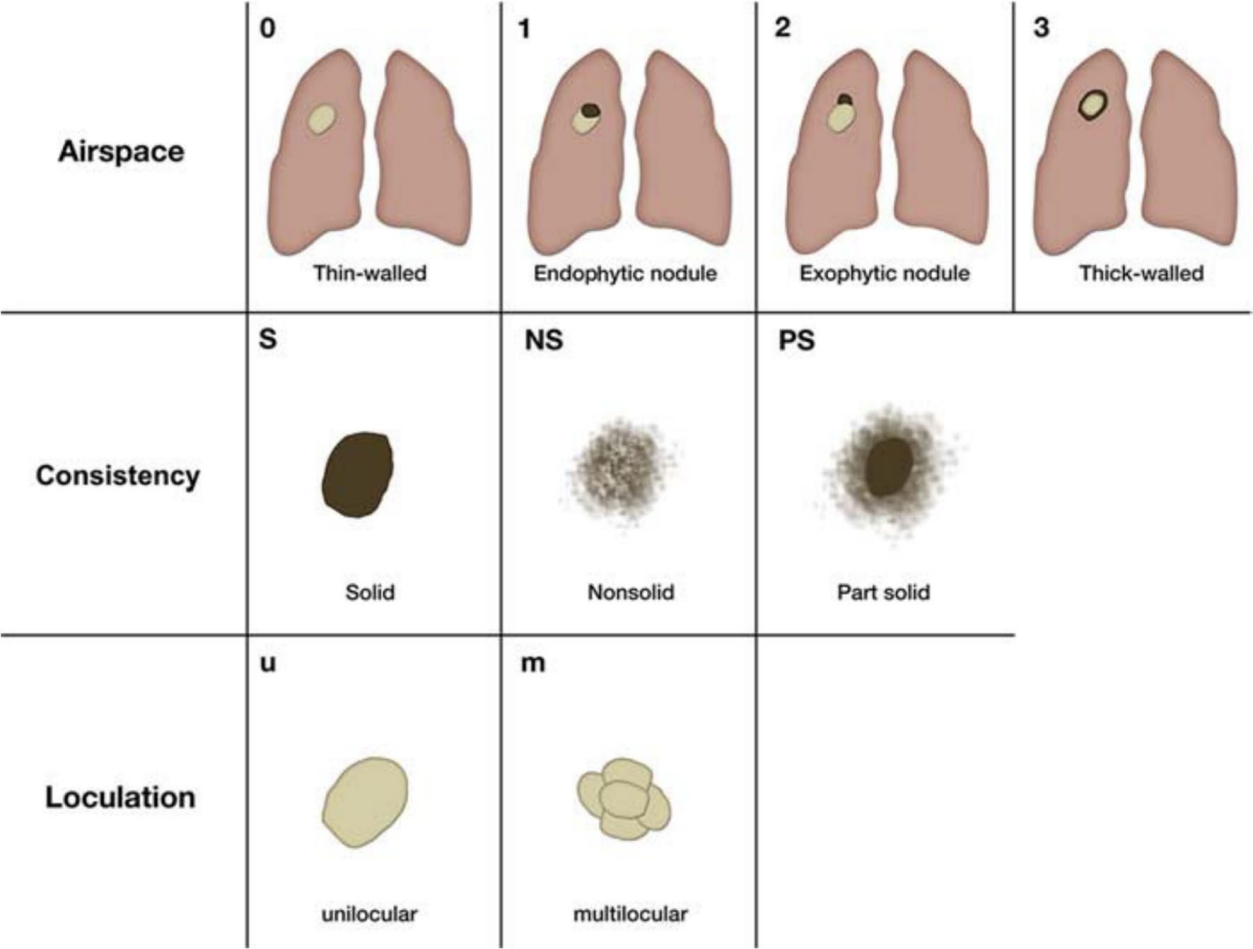
Courtesy of Florian J Fintelmann et al. (Ref. [59]). Classification scheme of lung cancers associated with cystic airspaces

*Cystic lesions.* An important and presumably under recognized morphology of (early) lung cancer is the cyst-related primary lung malignancy [64]. In the latest guidelines from the Fleischner Society, management of cystic lesions was not addressed, however the incidence of lung cancer associated with cystic spaces is not trivial, ranging from 1% to almost 4% [65]. Benign pulmonary cysts are usually characterized by thin walls (< 2 mm) and may result from previous infection or trauma, whereas features of malignancy include asymmetrical wall thickening, with development of endophytic or exophytic mural nodule and subsequent replacement of the cystic airspace by soft tissue [9]. The predominant histological correlate of cystic lung cancer is adenocarcinoma [65, 66]. The development of cystic areas within the lesion is believed to be related to a check-valve mechanism obstructing the small airways due to tumor growth [67]. Mascalchi et al. provided a classification of lung cancer associated with cystic airspaces in 4 types based on the wall thickness, the presence of endophytic or exophytic nodule, and the number of cystic areas [68], which was later modified by Fintelmann and colleagues by adding the consistency of the nodular component (Fig. 4) [65]. Based on this classification and on the TNM classification system for subsolid nodules, which considers only the solid component for staging [69], an adapted approach may be used for cystic lesion by measuring the extent of the nodular component and monitoring its growth over time, rather than considering the size of the air lucency component [70]. Moreover, the management of cystic lesions was incorporated in latest Lung-RADS criteria, which refer to lung cancer screening protocols, where lung nodules cannot be considered as incidental findings and does not apply to daily routine computed tomography [12]. Until an optimal follow-up strategy is defined for cyst-related lesions, some authors proposed a short-term follow-up at 3–6 months to evaluate rapid changes and, after exclusion of fast growth, a management similar to that of suspected subsolid lung nodules, considering that it is good clinical practice to intervene as soon as the aspect of the lesion changes and/or when targetable solid components are present, especially in patients without substantial comorbidity and with substantial life expectancy [64]. Hence, to avoid misinterpretation or delay in diagnosis of malignant pulmonary cyst lesions, increased awareness of this atypical lung cancer is urgently needed and a standardized management protocol has to be established.

#### Challenges related to subsolid nodules

Recent studies provided new insights on the natural history of subsolid nodules (SSN), considering their outcome on a long-term observation period and suggesting a longer follow-up period, especially considering that pure ground glass nodules may show a median follow-up of 9 years to grow [71], as well as analyzing the potential stage shift of early-stage adenocarcinoma based on volume doubling time (VDT) [72]. Moreover, it is worth considering that SSN showed a significantly higher prevalence in Eastern countries compared to Western countries, with Asian populations exhibiting a higher rate, particularly among females, raising concerns about overdiagnosis in lung cancer screening, potentially leading to unnecessary interventions [73].

Furthermore, it has been observed that certain subsolid nodules—referred to as heterogeneous—do not show visible solid components on the mediastinal window. This window is typically used to define the solid part of a nodule. However, these nodules do present a high-attenuation component on the lung window, which exceeds what is defined as ground-glass opacity (i.e., increased attenuation that does not completely obscure the underlying bronchial and vascular structures). Such nodules demonstrate an intermediate evolution between pure ground-glass nodules and true part-solid nodules [74, 75]. This characteristic can make the characterization and management of such nodules challenging. While awaiting new guidelines to clarify the appropriate approach, in our daily practice we tend to consider cautiously these nodules with high-attenuation components as part-solid.

#### Is suggested management reliable in clinical practice?

The last Fleischner Society guidelines for incidental pulmonary nodules management were published in 2017, namely more than 5 years ago. In 2022, Farjah and colleagues published a paper in which they show results from their retrospective study conducted over more than 5000 patients with incidentally discovered lung nodules, to verify whether Fleischner Society recommendations aligned with the goals of avoiding unnecessary follow-up in patients with < 1% risk of having lung cancer and pursuing further diagnostic evaluations in patients with > 1% probability of lung cancer [76]. Alignment between these goals and recommendations occurred in 51% of the study population, accounting for 91% of lung cancer cases, suggesting that it is appropriate to require further evaluations in patients at risk of lung cancer, but at the same time there is uncertainty about the ability of avoiding follow-up in those with low risk (< 1%). However, stratifying patients in high and low risk only considering their smoking status, that could be incomplete for such a purpose. Hence, further studies are needed to better understand the alignment between Fleischner Society guidelines and its explicit goals, and the suggested recommendations still represent the mainstay for the best management of incidental pulmonary nodules. Moreover, data suggest that a high number of patients are lost to follow-up of potentially malignant nodules, due to wide disparities in adherence to the guidelines among both radiologists and other involved physicians, such as pulmonologists, internists, and general practitioners. Therefore, structured nodule management programs, also with the implementation of automated tools, may be beneficial to increase adherence to guidelines, follow-up rates and lead to earlier-stage diagnoses [77].

## Interstitial lung abnormalities (ILAs)

ILAs are defined as the presence of nondependent abnormalities affecting more than 5% of any lung zone (upper, middle, and lower lung zones are demarcated by the levels of the inferior aortic arch and right inferior pulmonary vein) [3]. This definition is based exclusively on the radiological findings, but it is important to consider that, although ILAs and interstitial lung disease (ILD) represent two distinct entities, a subset of patients with ILAs are symptomatic and represent a mild stage of ILD, therefore the identification of such abnormalities on CT examination may have a clinical impact [3]. With the increasing number of performed chest CT scans, the detection of ILAs is becoming a common finding, especially in older patients, as described in recent cohort studies [78–81]. In the MESA Lung Study, where almost 14,000 cardiac CT scans were reviewed, the incidence of ILAs was estimated to be 13.1 cases per 1000 person-years [82]. The presence of ILAs is associated with worsened clinical outcomes and increased mortality compared to patients without ILAs at the initial CT scan [83]. Moreover, an association between the presence of ILAs and an increased incidence of lung cancer has been demonstrated [84, 85]. A recent meta-analysis showed that ILAs are associated with an elevated risk of overall mortality, respiratory-related deaths and with a notable increase in lung cancer and cancer treatment-related complications [86]. Hence, the early detection of ILAs may be crucial for the clinical course of these patients, and they should be always mentioned and correctly categorized in the radiological report.

### Risk factors for ILAs

Demographic, genetic and environmental are considered major risk factors associated both with prevalence and progression of ILAs [81]. Advanced age has been reported as a clear risk factor for ILAs [3, 80, 83, 87]. Each 10-year increase in age was associated with a 2.2 times increase in the odds of detecting ILAs [78]. Distinguishing the physiological changes of the lung in elderly patients from the presence of fibrotic alterations requiring clinical management is fundamental to avoid both over-monitoring and the risk of delayed therapy [88–90]; for instance, Copley et al. reported that a basal subpleural reticulations without traction bronchiectasis are often found on CT scans in asymptomatic elderly patients representing a normal aging-related parenchymal change [91]. Male sex remains an equivocal risk factor, as it has been associated with ILAs in some but not all studies [3, 87, 92, 93]. A genetic susceptibility for IPF, but also for ILAs and ILAs progression, has been associated with a common promoter polymorphism (rs35705950) in the gene encoding mucin 5B [81, 94, 95]. Environmental factors, such as cigarette smoking, air pollution, or other inhalational exposures, were found to be associated with an increased risk both for Idiopathic Pulmonary Fibrosis and ILA [3, 79, 88, 96–98].

### Subcategories of ILAs and risk factors for progression

**Fig. 5:**
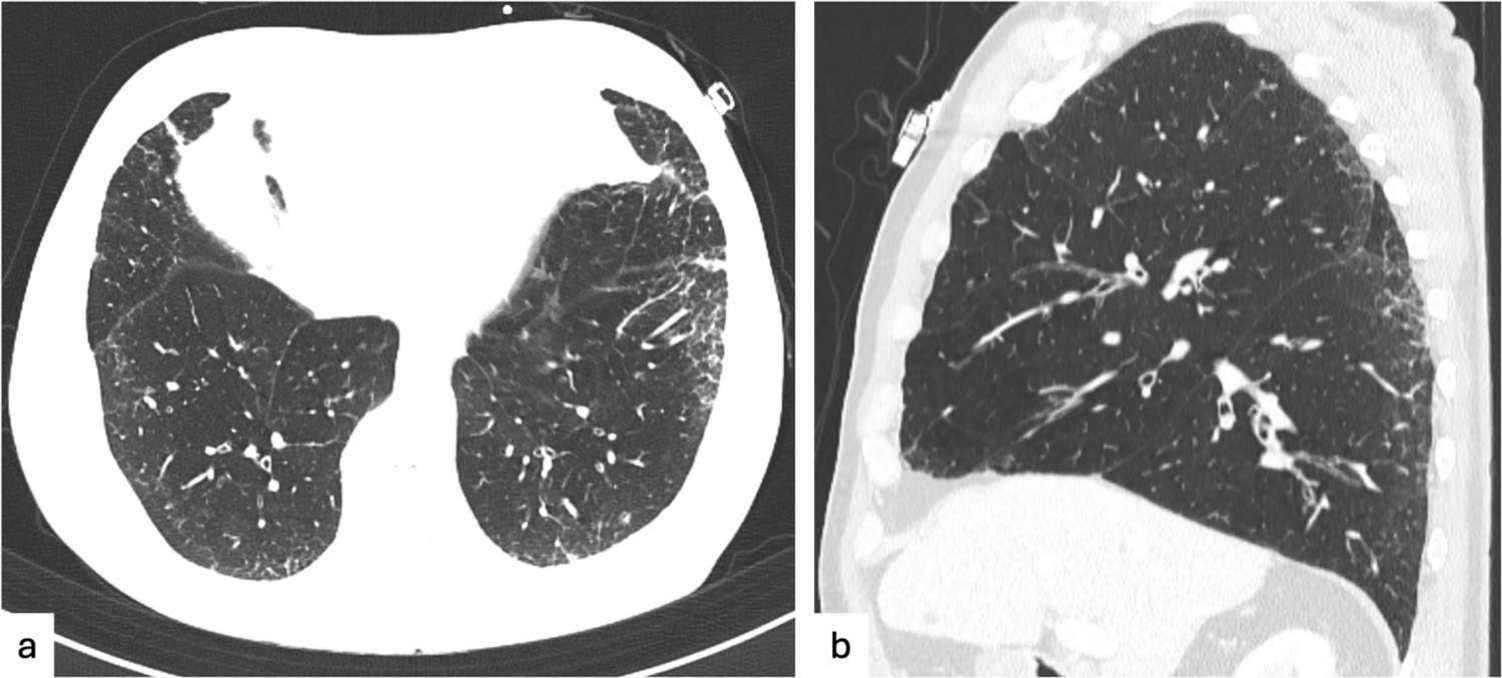
Seventy-three-year-old male patient who performed a cardiac CT scan, revealing subpleural bilateral reticulations and ground glass opacities, with a left lower lobe predominance, consistent with ILAs

**Fig. 6:**
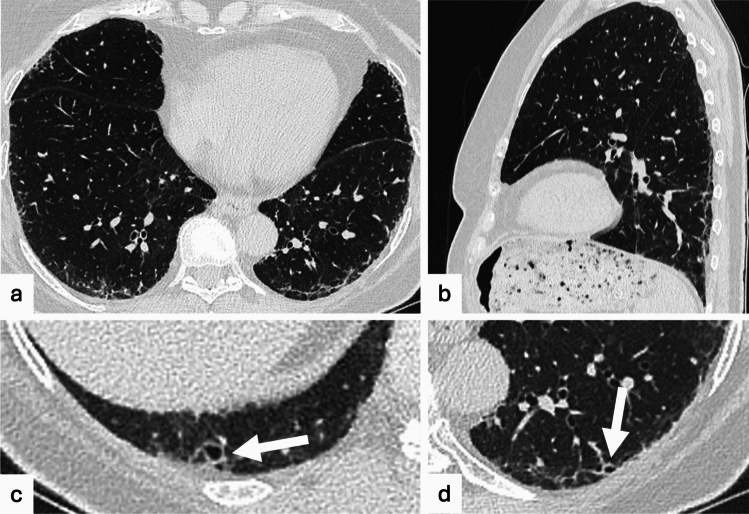
Seventy-one-year-old female. The chest CT scan, performed for a nodule seen on chest radiography, revealed bilateral peripheral reticulations with traction bronchiectasis (white arrows), classified as subpleural fibrotic ILAs

According to the 2020 position paper by the Fleischner Society, three subcategories have been described based on the distribution of interstitial abnormalities and the presence of fibrotic alterations: (1) ground-glass opacity and reticular opacities without a predominant subpleural localization; (2) ground-glass opacity and reticular opacities with a predominant subpleural localization without evidence of fibrosis (Fig. 5); (3) presence of features providing evidence of fibrosis, such as traction bronchiectasis, architectural distortion, and honeycombing [3] (Fig. 6). We can therefore summarize these categories as non-subpleural ILAs, subpleural non-fibrotic ILAs, subpleural fibrotic ILAs [92]. This classification is based on a study performed by Putman and colleagues, in which the imaging patterns of ILAs and the associated prognoses were investigated. In particular, subpleural and, more specifically, fibrotic ILAs were found to be associated with a greater likelihood of progression and with increased mortality, while non-subpleural ILAs are usually non-progressive [87]. Although reticulation, traction bronchiectasis, and subpleural fibrosis are considered as significant risk factors for progression, Park et al. demonstrated that the extent of fibrotic ILA was the only consistent independent risk factor for time to progression and, together with the presence of honeycombing, was an independent risk factors for time to progression to UIP [99].

### Pitfalls in ILAs

Correctly classifying imaging findings as ILAs may be insidious if not aware of the entities that are not considered ILAs (non-ILAs) and overlaps, which can sometimes be difficult to distinguish from ILAs, but this distinction is mandatory for the appropriate management of patients and to avoid unnecessary follow-up (see Table 3).

**Table 3:** Non-ILA and overlaps

Non-ILAs	
Dependent lung atelectasis Focal paraspinal fibrosis in close contact with osteophytes Apical cap Pleuroparenchymal fibroelastosis Interstitial oedema	

Overlaps	
Post-infection reticulation Scarring Usual interstitial pneumonia (UIP) Idiopathic pulmonary fibrosis (IPF) Desquamative interstitial pneumonia (DIP) Connective tissue disease-related interstitial lung disease (CTD-ILD) Sarcoidosis Pulmonary Langerhans cell histiocytosis (PLCH) Pneumoconiosis Fibrotic hypersensitivity pneumonitis Drug-related pneumonitis	

**Fig. 7:**
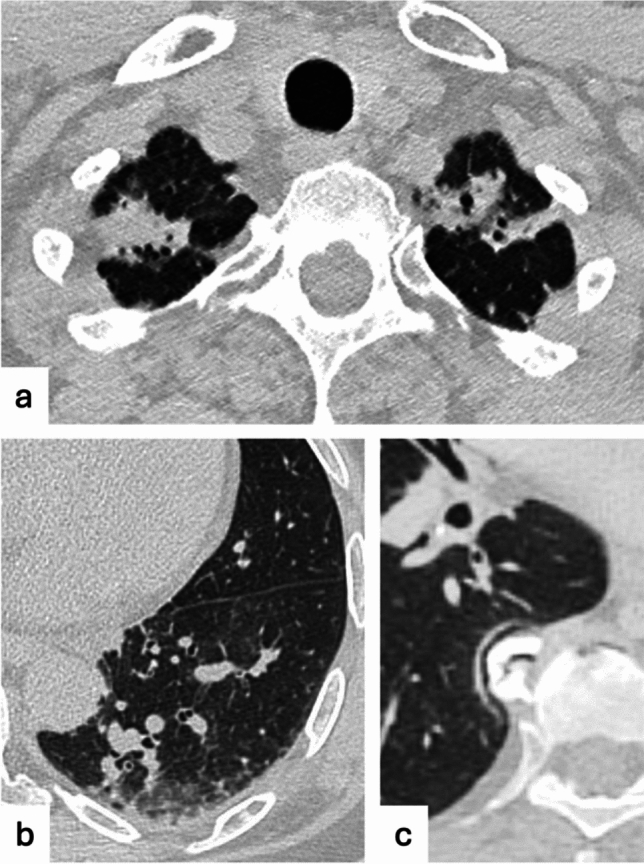
Examples of non-ILAs findings: apical scars (**a**), dependent lung subtle atelectasis (**b**), and focal paraspinal fibrosis close to an osteophyte (**c**)

Non-ILAs entities include ground-glass alterations in gravity-dependent zones, representing transient lung atelectasis, most commonly seen at posterior regions of lung bases; in such cases, an additional acquisition in prone decubitus may be resolutive [100]. Moreover, reticulations adjacent to vertebral osteophytes are typically seen in the right lower lobe and are caused by mechanical irritation of lung parenchyma, represent compressive atelectasis or fibrosis, but they rarely progress and therefore are not considered ILAs [92] (Fig. 7). Apical cap refers to lesions occurring at the apices of the lungs due to intrapulmonary and pleural fibrosis or caused by chronic ischemia resulting in hyaline plaques formation on the visceral pleura [101]. Sometimes it can be difficult to differentiate apical cap from early-stage pleuroparenchymal fibroelastosis, which is a rare condition characterized by pleural and subpleural parenchymal fibrosis and elastosis, usually involving the upper lobes [92]. However, both apical cap and pleuroparenchymal fibroelastosis are considered separate entities and not included in ILAs. Pulmonary interstitial edema is caused by the extravascular movement of fluid into the pulmonary interstitium and it is characterized by interlobular septal thickening, bilateral symmetrical ground-glass opacities, and consolidations in the advanced stages [102]. Clinical context, perihilar distribution of pulmonary edema, and the presence of pleural or pericardiac effusion when caused by decompensated heart failure can help differentiate it from ILAs.

Overlaps represent those CT findings that are similar to those of ILAs, although the disease may be differentiated from ILAs according to clinical background and symptoms. These include post-infection reticulation, scarring, UIP or IPF, desquamative interstitial pneumonia, ILD, ILD related connective tissue-related disease, sarcoidosis, pulmonary Langerhans cell histiocytosis, pneumoconiosis, fibrotic hypersensitivity pneumonitis, and drug-related pneumonitis [83]. It is worth emphasizing that in the context of high-risk patients, such as individuals with known connective tissue disease, familial ILD, and pneumoconiosis, treatment should be individualized according to their underlying condition, and the correct management needs to be clarified. When interstitial lung alterations are encountered on CT, possibly representing ILAs, radiologists should be aware of additional CT signs that may suggest overlaps rather than ILAs, such as esophageal dilatation (ILD related to systemic sclerosis) or calcified pleural plaques (that may be related to asbestosis) [103]. In such cases, referral to clinical evaluation or multidisciplinary discussion is fundamental to address the right diagnosis. In cases of overlapping between IPF and ILAs, further diagnostic testing is helpful for histological confirmation, including mini-invasive biopsy techniques such as transbronchial lung cryobiopsy (TBLC) [104].

### Assessment of ILAs extent

**Fig. 8:**
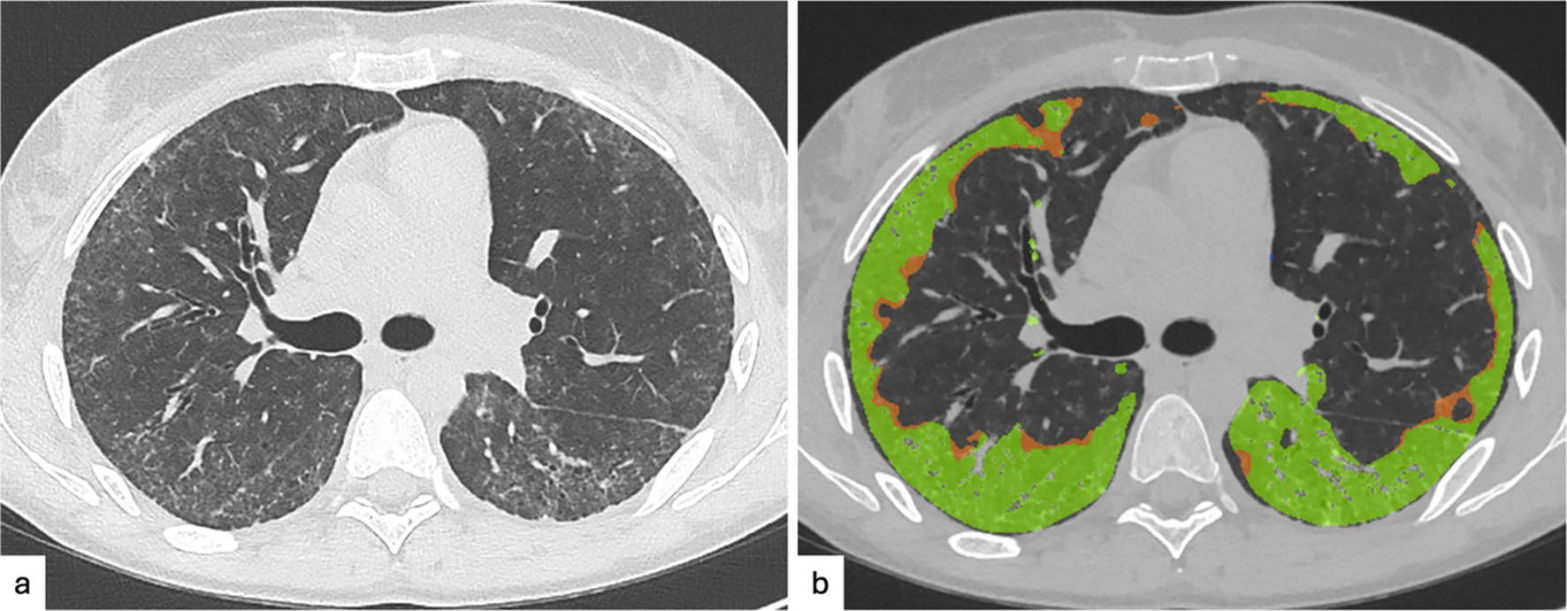
Forty-five-year-old patient with incidental fibrotic pulmonary alterations (**a**), representing fibrotic ILAs, with automated extent assessment (**b**), revealing 45% parenchymal involvement (42% ground-glass [green] + 3% reticulations [orange]). A multidisciplinary evaluation was required for this patient for suspected ILD

Automated assessment has been tested for quantifying the extent of ILAs on CT (Fig. 8). Kliment et al. compared visual and quantitative assessment for identification of ILAs, in a cohort of smokers enrolled in the COPDGene Study [78, 105]. CT findings were scored as “0”, no evidence of ILD; “1”, equivocal for ILD (focal or unilateral ground glass attenuation, focal or unilateral reticulation, and patchy ground glass abnormality affecting less than 5% of the lung; “2”, suspicious for ILD (nondependent ground glass abnormality affecting more than 5% of any lung zone, nondependent reticular abnormality, diffuse centrilobular nodularity with ground glass abnormality, honeycombing, traction bronchiectasis, non-emphysematous cysts, architectural distortion; “3”, ILD (bilateral fibrosis in multiple lobes associated to honeycombing and traction bronchiectasis in a subpleural distribution). The quantitative assessment was performed by calculating the extent of high-attenuation areas (HAAs) with an automated software [105]. HAAs are considered between − 600 and − 250 Hounsfield units (HU) (being the normal attenuation of lung parenchyma around -750 HU), reflecting the presence of ground-glass alterations, reticulations and excluding more dense abnormalities, such as consolidations, nodules or vascular structures [3, 83, 106]. Despite high-density regions have been associated with increased serum concentrations of inflammatory biomarkers and higher mortality, the detection methods based on classification using lung density alone appear to be not predictive of ILAs defined by visual assessment [105]. This phenomenon can be explained by the fact that there are factors affecting lung density measurements, such as inadequate inspiration, obesity, pulmonary atelectasis, and scanner variation [107]. However, in a Korean Lung Cancer Screening Program, a quantitative deep learning-based texture analysis showed 100% sensitivity and 99% specificity for detecting ILAs with use of a 1.8% of lung parenchyma as threshold [108]. In another studies [99, 109] software dedicated to ILAs analysis were adopted: Kim et al., with a deep learning-based system, identified ILAs despite the subtle disease extents and the application of various CT protocols, with a 68% sensitivity and 93% specificity, on applying at least 5% of lung abnormalities extent per zone and 84.8% sensitivity and 92.4% specificity using an adjusted area threshold of 3.6% [109]. Park et al. evaluated the impact of ILAs extent on the risk of progression [99]. These methods usually offer a more reproducible method for the detection and the assessment of progression of ILD compared to visual analysis [105].

### Management of ILAs and clinical implications

**Fig. 9:**
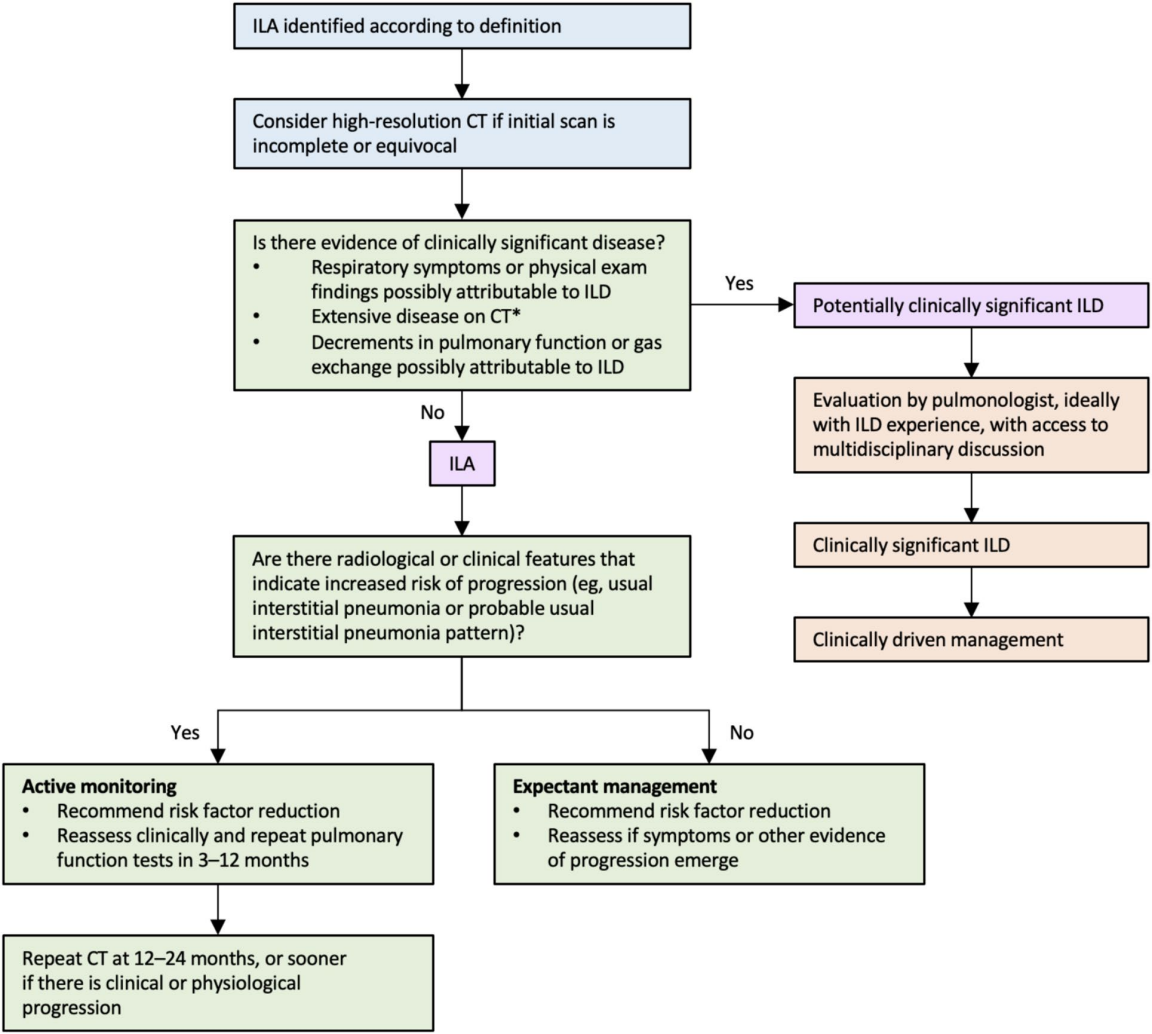
Proposed management for interstitial lung abnormalities detected on chest CT. Adapted with permission from reference 4. Action items for the radiologist are in blue, action items for the treating physician or pulmonologist are in green, and action items for a pulmonologist, ideally with ILD experience, are in orange. ILAs = interstitial lung abnormalities. ILD = interstitial lung disease. *Non-trivial abnormalities present in three or more lung zones (above the bottom of the aortic arch, between the aortic arch and top of the inferior pulmonary vein, and below the inferior pulmonary vein)

There is still poor evidence supporting a precise management plan for ILAs, however a scheme (Fig. 9) has been proposed by the Fleischner Society, based on the available literature and the consensus clinical opinion [3]. Once the suspicion of ILAs is made on the basis of chest CT features, the first step consists in distinguishing patients with clinically significant ILD, who need specific evaluation and management by pulmonologist, from those who are at risk of developing the disease. For instance, early ILD includes pre-clinical (individual at risk of ILD) and subclinical ILD (individuals not at risk for ILD) patients, in whom ILD has been ascertained according to guidelines, thus not considered ILAs [88]. Once ILD is excluded, patients can be divided into those at higher risk of progression to ILD and those at lower risk, based on radiological and clinical risk factors. Individuals with at least one risk factor are considered at higher risk, whereas those without such risk factors are at lower risk [83]. Both high-risk and low-risk groups should be informed and recommended of reducing modifiable risk factors such as cigarette smoking. The high-risk group may undergo an active monitoring consisting in pulmonary function testing in 3–12 months and CT at 12–24 months or earlier if patients develop respiratory symptoms or show impaired pulmonary function. The low-risk group is advised to return for reassessment if they develop any respiratory symptoms or other signs of progression [3]. However, Park et al. suggested a 3-year CT scan follow-up to monitor radiologic progression, except for high-risk patients [99]. It is worth considering that patients undergoing lung surgery, chemotherapy, or radiation therapy are at risk of disease exacerbation and rapid progression, therefore clinicians should be aware that ILAs may represent an important comorbidity in these patients [110–112]. In addition, as positive pressure ventilation may cause acute respiratory distress, a low-volume, low-pressure ventilatory approach should be applied in patients with ILAs requiring mechanical ventilation [3, 83].

It is worth considering that underestimation or misdetection of ILAs may be relevant for clinical implication of selected patients. In a recent analysis conducted by Sverzellati and colleagues over more than 20,000 patients with both abdominal and thoracoabdominal CT scans, ILAs were found in 1.7% of patients, with 1% showing fibrotic features [113]. Despite their association with increased mortality, 43.9% of ILAs were not reported by radiologists and fibrotic ILAs significantly increased the risk of death from respiratory cause, emphasizing the need for greater awareness, systematic reporting, and follow-up of ILAs, even when detected incidentally on non-chest CTs.

### What could change about ILAs

To date, radiologically, ILAs have been classified in three categories based on imaging appearance (non-subpleural, subpleural non-fibrotic, subpleural fibrotic) to identify high-risk patients, taking also into account some additional features (such as UIP or probable UIP pattern). However, fibrotic extent seem to be also a risk factor for ILAs progression [99]. Therefore, we expect that ILAs classification and management will be implemented on the basis of more elements, both clinical and radiological. Moreover, ILAs may be encountered incidentally in abdominal or cardiac CT scan, but no recommendation about their management in such cases has been suggested. It may be appropriate to address patients to clinical evaluation and, if indicated, to perform a supplemental acquisition of the chest; however, further analyses are needed in this context.

## Conclusions

Pulmonary nodule and ILAs represent, respectively, focal and diffuse incidental findings that may be discovered on chest CT, for which proper management is essential for a precise diagnostic and therapeutic workflow of patients.

Size remains the leading characteristic for nodule’s management, however in particular cases other factors must be considered to ensure a complete risk assessment of nodules. For instance, lung nodule malignancy risk is influenced by other additional features, such as shape, location, and growth rate. Larger, spiculated nodules in the upper lobes are more likely to be cancerous. Subsolid nodules grow slowly and could require extended follow-up. Other risk enhancers include emphysema, pulmonary fibrosis, smoking history, age, family history, and certain demographic traits like race and sex. While smoking remains the strongest known risk factor, exposure to other inhaled carcinogens like asbestos and radon also contributes. Current guidelines recommend using clinical models, such as those from the American College of Chest Physicians, to classify patients into low, intermediate, or high risk to guide monitoring and management decisions. Therefore, we illustrated possible risk factors which help identify nodules with potential risk of malignancy, such as intrinsic nodule characteristics, aspects related to lung parenchyma and to the patient’s history. Moreover, on the basis of current guidelines for incidental lung nodules management, we tried to clarify dubious scenarios as well as highlight where the correct management of incidentally discovered pulmonary nodules is not fully established, yet. Besides the issues concerning management of certain types of nodules in different scenarios, the main challenge is represented by the fact that a high number of patients are lost to follow-up, missing the chance for early diagnosis of potentially malignant nodules. Reasons may be attributed to low adherence to the existing guidelines, lack of clear communication and patient tracking, thus structured nodule management programs, also with the implementation of AI tools, may be beneficial to increase follow-up rates and lead to earlier-stage diagnoses.

On the other hand, the increasing clinical relevance of ILAs has led the Fleischner Society to release a proposal for the assessment and management of this entity, which is in evolution. However, the detection, correct identification, and the distinction of ILAs from imaging pitfalls resembling these alterations is crucial for addressing patients to further follow-up or to avoid unnecessary clinical or radiological investigation. In this article, we provided elements of recent evidence supporting and helping the radiologist in the recognition and management of ILAs, addressing further potential developments in their evaluation. Moreover, current management of ILAs is mainly based on expert opinion and limited evidence. Guidelines recommend follow-up based on risk factors (clinical, drug-related, and radiological). Invasive tests like cryobiopsy can help confirm diagnoses in selected patients, but decisions should be individualized. A significant concern is that early IPF may be hidden among fibrotic ILAs, suggesting a need for better diagnostic tools and biomarkers. While early treatment may help, strong clinical evidence is lacking. Therefore, a call for improved ILAs characterization, better diagnostic pathways, and ultimately, a move toward precision medicine for earlier and more effective intervention is needed.

## Data Availability

Data sharing is not applicable to this article as no new data were created or analyzed in this study.
